# Evaluation of the Acute flaccid paralysis surveillance indicators in Zambia from 2015–2021: a retrospective analysis

**DOI:** 10.1186/s12889-023-17141-1

**Published:** 2023-11-12

**Authors:** Barnabas Bessing, Edward A. Dagoe, Deborah Tembo, Alice Mwangombe, Muzala K. Kanyanga, Fadinding Manneh, Belem B. Matapo, Patricia M. Bobo, Musole Chipoya, Victor A. Eboh, Princess L. Kayeye, Penelope K. Masumbu, Chilweza Muzongwe, Nathan N. Bakyaita, Delayo Zomahoun, Jude N. Tuma

**Affiliations:** 1 World Health Organization Country Office, Lusaka, Zambia; 2 United States Centers for Disease Control and Prevention, Atlanta, USA; 3 Zambia National Public Health Institute, Lusaka, Zambia; 4 World Health Organization Africa Regional Office, Congo Brazzaville, Republic of Congo; 5 Ministry of Health Headquarters, Lusaka, Zambia; 6 McKing Consulting Corporation, Lusaka, Zambia; 7 Taskforce for Global Health, Decatur GA, USA; 8 World Health Organization Headquarters, Geneva, Switzerland

**Keywords:** Acute Flaccid Paralysis, Indicators, Poliomyelitis, Surveillance, Zambia

## Abstract

**Background:**

The resurgence of poliovirus infection in previously polio free regions and countries calls for renewed commitment to the global polio eradication efforts including strengthening of Acute Flaccid Paralysis (AFP) surveillance systems. Zambia is one of the countries substantially at risk for the importation of poliovirus infection from neighbouring countries including Malawi, Mozambique, and Democratic Republic of the Congo (DRC). This study describes a seven-year AFP surveillance, assesses the surveillance indicators, and highlights areas for improvement.

**Methods:**

We conducted retrospective analysis of the routinely collected AFP surveillance data from January 2015 to December 2022. The AFP surveillance indicators performance was assessed using the World Health Organisation’s recommended minimum AFP surveillance indicators performance.

**Results:**

Cumulatively, a total of 1715 AFP cases were reported over the study period. More than half, 891 (52%) of reported cases were aged < 5 years with 917 (53.5%) of males. More than half, 1186 (69.2%) had fever at onset, 718 (41.9%) had asymmetric paralysis and 1164 (67.9%) had their paralysis progressed within 3 days of onset. The non-polio AFP rate ranges from 3.4 to 6.4 per 100,000 children < 15 years old and stool adequacy ranging from 70.9% to 90.2% indicating sensitive surveillance with late detection of cases. The percentage of cases with early stool collection, timely transportation was above the World Health Organisation (WHO) minimum of 80% but with declining proportion of stools arriving in the laboratory in optimal condition. Completeness of 60-days follow-up evaluation was suboptimal ranging from 0.9% to 28.2%.

**Conclusion:**

The AFP surveillance system in Zambia is doing well. However, additional efforts are needed to improve early detection of cases; stool sample collection, transportation and monitoring to ensure arrival in good condition in the laboratory; and improve 60-days follow-up evaluation for evidenced-based classification of inadequate AFP cases and proper care.

## Background

Poliomyelitis (Polio) is a contagious viral infectious disease caused by any three serotypes (1, 2 and 3) of poliovirus [1–3]. Poliovirus is transmitted via the faecal-oral route with more than 90% of its infections being subclinical, and the rest present with aseptic meningitis or abortive polio (self-limiting illness of fever, headaches, sore throat, malaise, and myalgia) [2, 4]. A small proportion (< 1%) of poliovirus infections leads to neuronal destruction and irreversible paralysis that initially manifest as Acute Flaccid Paralysis (AFP) [5]. While poliovirus infection can occur at any age, children below fifteen years are most vulnerable with most infections occurring in children under 5-years [1, 3]. There is no cure for polio, but it can be prevented via safe polio vaccination [1].

Polio virus is an enterovirus which has no extra human reservoir and as a result can easily be eradicated [5]. In view of this, the Global Polio Eradication Initiative (GPEI) was inaugurated in 1988 at the World Health Assembly to eradicate polio [6]. The GPEI since its commencement has reduced the number of wild polio cases worldwide by 99% [7] via increased population immunity and improved surveillance using four main strategies: strong routine immunisation using polio vaccine; supplementary polio immunisation activities; mop-up polio immunisation in hard-to-reach and low coverage areas; and robust AFP surveillance and reporting [8].

Acute Flaccid Paralysis (AFP) is a clinical syndrome characterised by a sudden onset of flaccid (reduced muscle tone) weakness of a limb in a child < 15 years old [9]. AFP mimics the clinical presentation of many diseases associated with enterovirus infections including poliomyelitis, Guillain Barre syndrome, transient paralysis, transverse myelitis, and traumatic neuritis [1–11]. The World Health Organization (WHO) therefore recommended a robust AFP surveillance system where all AFP cases are investigated, stool sample collected, and tested for poliovirus in a WHO accredited laboratory as the gold standard for monitoring poliovirus infection [11–13]. In a sensitive AFP surveillance system, the absence of poliovirus infection detection over time implies the absence of poliovirus transmission and may inform decision on certification of polio eradication [11, 14].

Zambia was declared polio free in 2005 after its last confirmed indigenous wild poliovirus in 1995. The country has since used its AFP surveillance system and expanded program on immunisation (EPI) to maintain its polio-free status. However, in early 2022, wild poliovirus (WPV) infections remerged in South-Eastern Africa (in Malawi and Mozambique) eighteen months after the continent was declared indigenous wild poliovirus-free [15, 16]. These viruses that appeared to have been in circulation in the region for almost three years are genetically linked to poliovirus in Pakistan [15, 16]. These wild polio outbreaks implied possible wider regional missed circulation of the virus mainly driven by low surveillance sensitivity and low population immunity that could not prevent and interrupt the virus transmission [1, 17]. Zambia shares borders with eight different countries including Malawi and Mozambique where there are high population movements and therefore is at higher risk of importation and transmission of wild poliovirus. A longitudinal evaluation of the AFP surveillance system in Zambia may provide relevant evidence to support informed decisions towards improving the surveillance system. This paper therefore evaluates the seven-year (2015–2021) performance of the AFP surveillance system in Zambia and identifies areas that require improvement for action to maintain a polio-free Zambia.

## Methods

### Study setting and design

Zambia is a landlocked country in the Southern African continent with a total surface area of 752,612 square kilometres. The country shares common boundary with Democratic Republic of the Congo in the north, Tanzania in the northeast, Malawi in the east, Mozambique in the southeast, Zimbabwe and Botswana in the south, Namibia in the southwest, and Angola in the west. Since 2019, Zambia is administratively divided into 10 provinces with 116 districts. The country has a subtropical climate characterized by 3 main seasons: the rainy season (November to April), which is warm and wet; the cold season (May/June to October/ November), which is dry and cold, and the hot season (September to October), which is dry and hot. In this study, we conducted a retrospective descriptive analysis of routinely collected AFP surveillance data from January 2015 to December 2021. All AFP cases that were reported nationwide during this period were included.

### Acute flaccid surveillance system in Zambia

Acute Flaccid Paralysis (AFP) Surveillance activities started in the capital Lusaka in 1993 and later scaled up nationwide in August 1998. In the surveillance system, an AFP is defined as “any child < 15 years of age presenting with sudden (acute) onset of flaccid (floppy) paralysis or muscle weakness, or any person of any age with paralytic illness whom a clinician suspects poliomyelitis. The AFP case-based surveillance has been adopted as the gold standard and is being conducted via the Integrated Disease Surveillance System (IDSR) by the Ministry of Health (MOH) and coordinated by the Zambia National Public Health Institute (ZNPHI) surveillance and disease control department. There are established surveillance structures at all levels of the health service delivery with community health volunteer/informant at community level, surveillance focal person at health facility level, disease surveillance officer at district level, and provincial surveillance officer at provincial level.

**Fig. 1:**
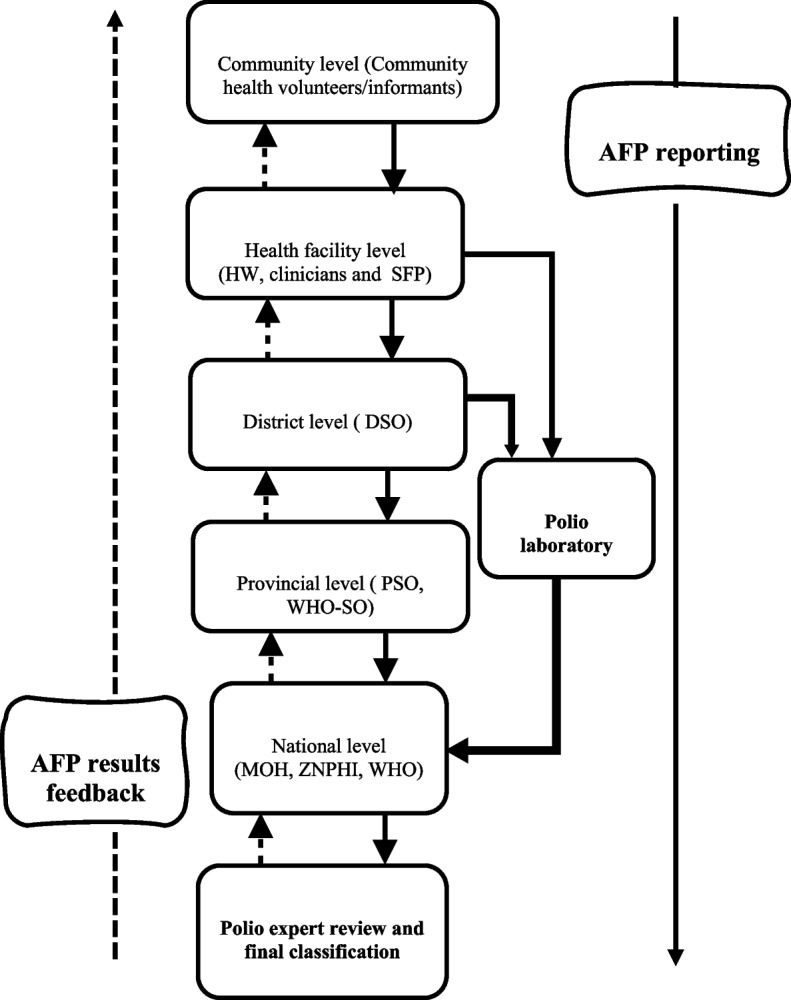
Flow chart of AFP surveillance system in Zambia. HW: Health Worker; SFP: Surveillance Focal Person; District Surveillance Officer; Provincial Surveillance Officer; World Health Organisation Surveillance Officer; MOH: Ministry of Health; ZNPHI: Zambia National Public Health Institute

Figure 1 represents the flow of information and feedback mechanism of the AFP surveillance system from the community to national levels. The primary purpose of the AFP surveillance system is to ensure no polio case goes undetected by identifying, reporting, investigating, and collecting stool samples from all AFP cases to test for poliovirus in the national WHO polio accredited laboratory. All AFP cases identified by the community health volunteer or informants (traditional healers, religious leaders) or health facility staff (clinicians or facility focal persons) are reported to the districts disease surveillance officer (DSO) for further verification and validation. The DSO support the facility surveillance focal person (SFP) to fill the AFP case investigation form, collect two adequate stool samples with adequate documentation and transport using the required optimal temperature to the national polio laboratory at the University Teaching Hospital (UTH) in Lusaka. The DSO ensures the AFP sample results are communicated back to the facility and community. At the provincial level, the provincial surveillance officer, or the WHO surveillance officer assigned to that province or the field epidemiology training program trainee under the ZNPHI supports the DSO to conduct AFP case validation and 60-days follow-up evaluations and reports to the national level. At the national level, the ministry of health MOH in collaboration with the ZNPHI and WHO coordinate the reporting and provide the needed information for the national polio expect committee (NPEC) to conduct the final classification of all reported AFP cases.

### Measures

#### Demographic and clinical variables

During AFP case investigation, information including demographic and clinical and vaccination history is collected. In this study data on age (months), sex (male or female), polio vaccination status (vaccinated or not), number of doses received from either the routine or SIAs if vaccinated, fever at onset of paralysis (Yes or No), progression of paralysis within 3 days after onset of paralysis (Yes or No), and nature of the paralysis (asymmetric or symmetric) were extracted from the existing polio database for analysis.

#### Surveillance performance indicators

The WHO established minimum performance indicators that are used to assess and maintain the quality of AFP surveillance system [13, 18, 19]. In this study, we evaluated the performance of the surveillance system using the WHO-specified indicators as follows:

- *Non-polio acute flaccid paralysis (NPAFP) rate*: this refers to number of discarded AFP cases < 15 years that are not due to polio per 100,000 population age < 15 years. The recommended target is ≥ 2 per 100,000 population of < 15 years in endemic regions and outbreak affected areas. This indicates that the AFP surveillance system is sensitive [13, 18, 19].
- *Stool adequacy*: This refers to the proportion of AFP cases with two adequate stool samples (≥ 8 g) collected in ≥ 24 h apart and within 14 days after onset of paralysis, transported on ice/frozen packs and received in a WHO-accredited laboratory in a good condition. The recommended target is ≥ 80% [18, 19].

Subnational composite index (achieving both core indicators):

This refers to the proportion of districts with ≥ 100,000 children < 15 years old that achieved both the NPAFP rate of ≥ 2/100,000 < 15 years population and stool adequacy of ≥ 80% targets. The recommended target is ≥ 80% [18, 19].

- *Timeliness of AFP case notification*: This refers to the proportion of AFP cases reported to the district or facility level surveillance team within 7 days of onset of paralysis. The recommended standard is ≥ 80% [13, 18, 19].
- *Timeliness of AFP case investigation*: this refers to the proportion of reported AFP cases investigated by the surveillance team within 48 h of notification. The recommended standard is ≥ 80% [13, 18, 19].
- *Timeliness of stool samples shipment*: This refers to the proportion of AFP cases stool samples arriving in good condition at a WHO-accredited laboratory and within 3 days of collection of second stool sample. The recommended target is ≥ 80% [13, 18, 19].
- *Stool condition*: the refers to the proportion of stool samples that arrive in the laboratory in the temperature range of + 2 °C to + 8 °C, with no spillage/desiccation and with the appropriate documentation. The recommended target is ≥ 80% [13, 18, 19].
- *Completeness of follow-ups*: this refers to the proportion of AFP cases with a follow-up evaluation for residual paralysis completed ≥ 60 days and ≤ 90 days after onset of paralysis. The recommended target is ≥ 80% [13, 18, 19].

### Data source and analysis

We extracted this study data from the WHO Polio Information System (POLIS) 2022 dashboard. The POLIS platform allows countries to load, clean and harmonize polio surveillance data at sub-national, national, and regional levels for decision making. Data from January 2015 to December 2021 was extracted in a Microsoft comma separated values (Ms csv) file formats which included all key demographic, clinical, vaccination, and polio surveillance variables. The data was then compared with the country level Access database for consistency and quality checks.

We then conducted descriptive statistical analysis using Microsoft Excel (version 2021) spreadsheet for key polio surveillance indicators. Maps were developed using Quantum Geographical Information System (QGIS) version 2.18.10 to visualise the key surveillance indicators performance at the provincial and district levels. Findings of the study were presented using tables, charts, and maps. All outcome parameters were based on the WHO recommended performance indicators of AFP surveillance [13, 18, 19].

## Results

### Demographic and clinical characteristics

**Fig. 2:**
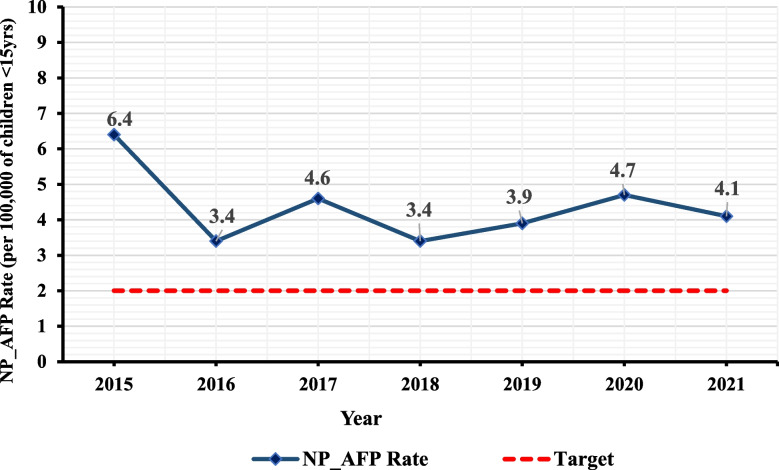
Zambia annualized NPAFP rate from 2015–2021

Cumulatively, 1,715 AFP cases were reported in the country between January 2015 and December 2021 with an average age of 5.5 years (Fig. 2). Of all cases, 891 (51.9%) were under five years, 917 (53.5%) were male and majority (53%) had received three or more oral polio vaccine (OPV) doses. In terms of clinical history, 1,186 (69.2%) of all cases reported fever at onset of paralysis, 718 (41.9%) had asymmetric paralysis, and 1164 (67.9%) had their paralysis rapidly progressed within three days of onset (Table 1).

**Table 1:** Demographic and clinical characteristics of reported AFP cases in Zambia from 2015–2021

Characteristics	Year of reporting						
	**2015**	**2016**	**2017**	**2018**	**2019**	**2020**	**2021**
Age (in months), mean (SD)	69 (65.6)	66 (63.8)	62 (64.1)	74 (66.7)	67 (48.5)	57 (47.3)	67 (74.2)
Age categories, n (%)							
< 5 years	111 (48%)	125 (53%)	163 (57%)	96 (49%)	105 (45%)	172 (59%)	119 (53%)
5–15 years	113 (49%)	103 (44%)	117 (41%)	95 (43%)	123 (53%)	121(41%)	101(45%)
> 15 years	7 (3%)	7 (3%)	7 (2%)	6 (3%)	4 (2%)	1 (0.3%)	6 (3%)
Sex, n (%)							
Female	103 (46%)	108 (47%)	137 (48%)	95 (48%)	105 (46%)	147(51%)	103 (48%)
Male	121 (54%)	123 (53%)	148 (52%)	103 (52%)	125(54%)	143(49%)	112 (52%)
Vaccination history							
Zero dose	6 (4%)	3 (2%)	5 (3%)	0 (0%)	1 (1%)	2 (1%)	9 (7%)
1–2 doses	24 (16%)	26 (17%)	23 (12%)	15 (14%)	23 (15%)	29 (15%)	23 (17%)
3 + doses	122 (80%)	128 (82%)	162 (85%)	94 (86%)	128 (84%)	169 (85%)	101 (76%)
Clinical history, n (%)							
Fever at onset of paralysis (yes)	161 (73%)	227 (95%)	205 (75%)	141(71%)	151 (65%)	172 (59%)	129 (56%)
Paralysis progress ≤ 3 days (yes)	159 (72%)	233 (98%)	180 (75%)	138 (70%)	153 (66%)	171 (58%)	130 (57%)
Asymmetrical paralysis (yes)	89 (40%)	90 (38%)	111 (46%)	95 (48%)	113 (49%)	125 (43%)	95 (42%)

*AFP surveillance performance*

Table 2 present a summary of the AFP surveillance indicators evaluated in this study. The average national annualised non-polio AFP detection rate was 4.4 per 100,000 population < 15 years of age, indicating the surveillance system is sensitive at the national level. Over the evaluation period, the country’s AFP detection rate was consistently above the recommend minimum of ≥ 2.0 cases per 100,000 (Fig. 2) ranging from 6.4 cases per 100,000 population under 15 years in 2015 to 4.1 cases per 100,000 population under 15 years in 2021. This was similar in all 10 provinces with annualised rate ranging from 5.0 cases per 100,000 population under 15 years in 2015 to 4.0 cases per 100,000 population under 15 years in 2021.

**Table 2:** AFP surveillance indicators performance in Zambia from 2015–2021

Indicators	Target	2015	2016	2017	2018	2019	2020	2021
Number of AFP cases reported	-	234	239	287	199	232	294	230
Annualised NPAFP rate^a^	** ≥ 2**	6.4	3.4	4.6	3.4	3.9	4.7	4.1
% Stool adequacy	** ≥ 80%**	86.8%	88.3%	90.2%	85.9%	84.5%	71.1%	70.9%
% achieved both core indicators	** ≥ 80%**	20%	20%	40%	26%	25%	22%	13%
% Of cases notified ≤ 7 days of onset of paralyses	** ≥ 80%**	77.9%	73.7%	74.6%	71.7%	70.1%	58.5%	57.8%
% Of cases investigated ≤ 48 h of notification	** ≥ 80%**	87.6%	96.7%	91.6%	91.0%	91.4%	92.9%	97.4%
% Of specimen arriving in the lab ≤ 3 days	** ≥ 80%**	93.6%	95.0%	90.6%	97.0%	94.0%	94.5%	90.4%
% Arriving in lab in good condition	** ≥ 80%**	85.5%	88.7%	90.2%	84.4%	82.3%	71.3%	70.0%
% Of AFP cases with 60-days follow-ups	** ≥ 80%**	13.7%	9.6%	10.5%	18.1%	0.9%	28.2%	15.2%

*AFP* Acute Flaccid Paralysis, *NPAFP* non-polio acute flaccid paralysis

^a^The rate is per 100,000 population of children under 15 years of age

**Fig. 3:**
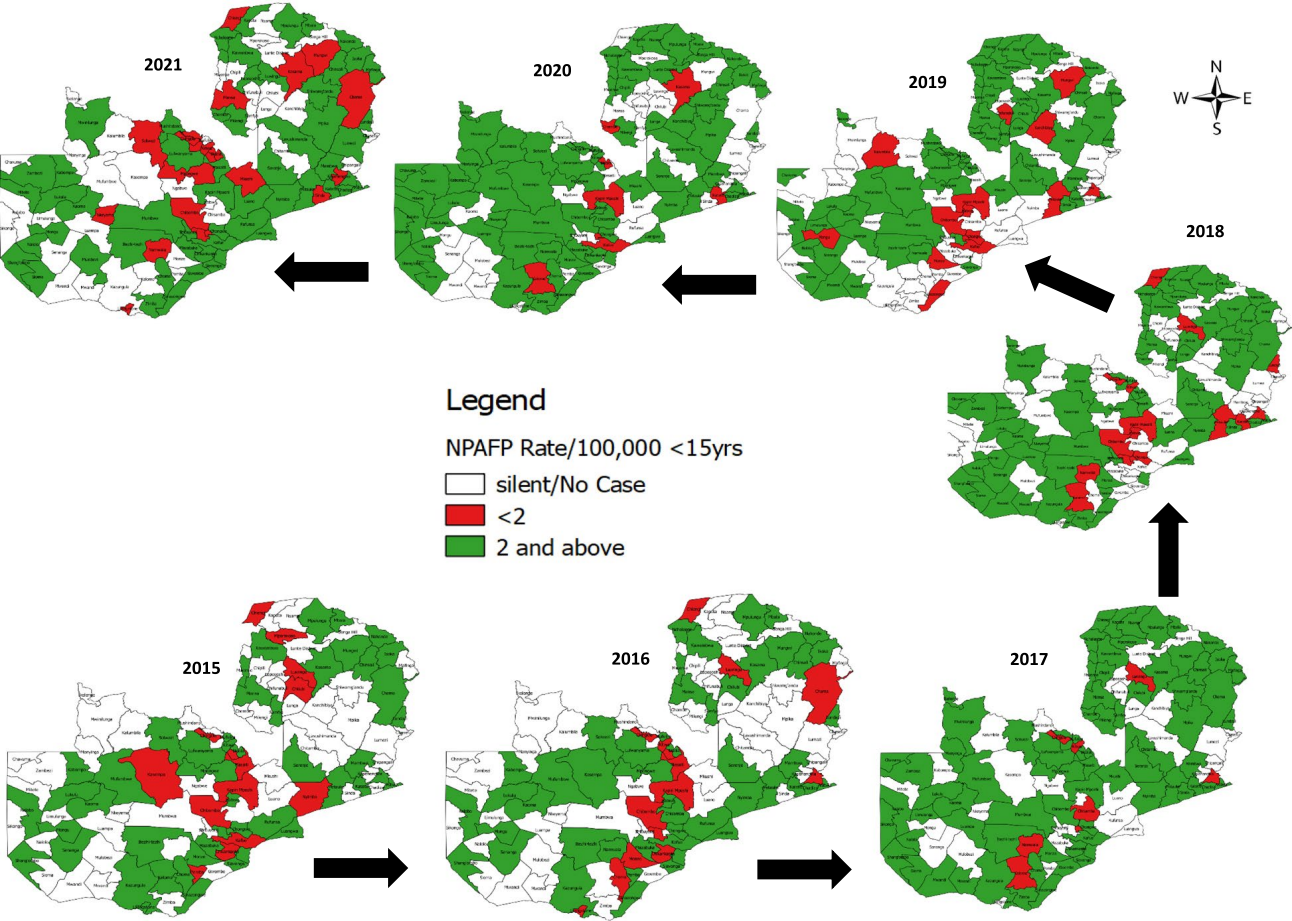
Pattern of non-polio acute flaccid paralysis rates by districts in Zambia from 2015–2021

However, there are few gaps at the district level as few districts have not achieved this indicator with some remaining silent for some period (Fig. 3). For instance, only 9% (10) of districts, all rural, consistently achieved a NPAFP rate of ≥ 2/100,000 < 15 years population over the seven-year period and this increased slightly to 18% (21) in the last three years (2019–2021) consisting of 86% (18) and 14% rural and urban districts, respectively. Similarly, about 4% (5) of the districts, all rural, consistently did not achieve this indicator but this proportion increased to 9% (10) in the last three years (2019–2021) consisting of 90% (9) and 10% (1) rural and urban districts, respectively.

**Fig. 4:**
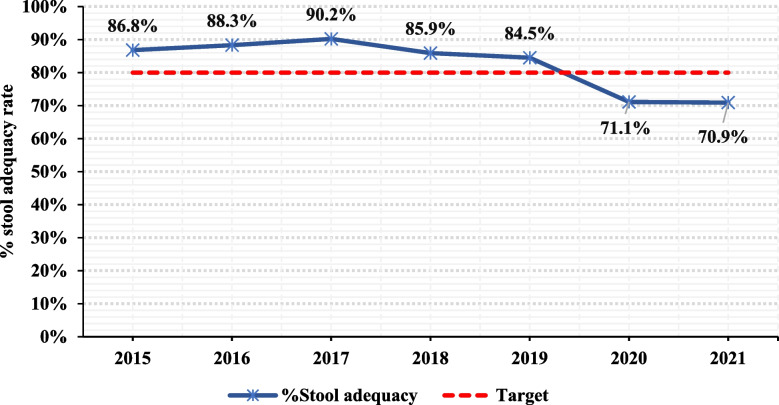
Pattern of stool adequacy rate performance of AFP cases in Zambia from 2015–2021

**Fig. 5:**
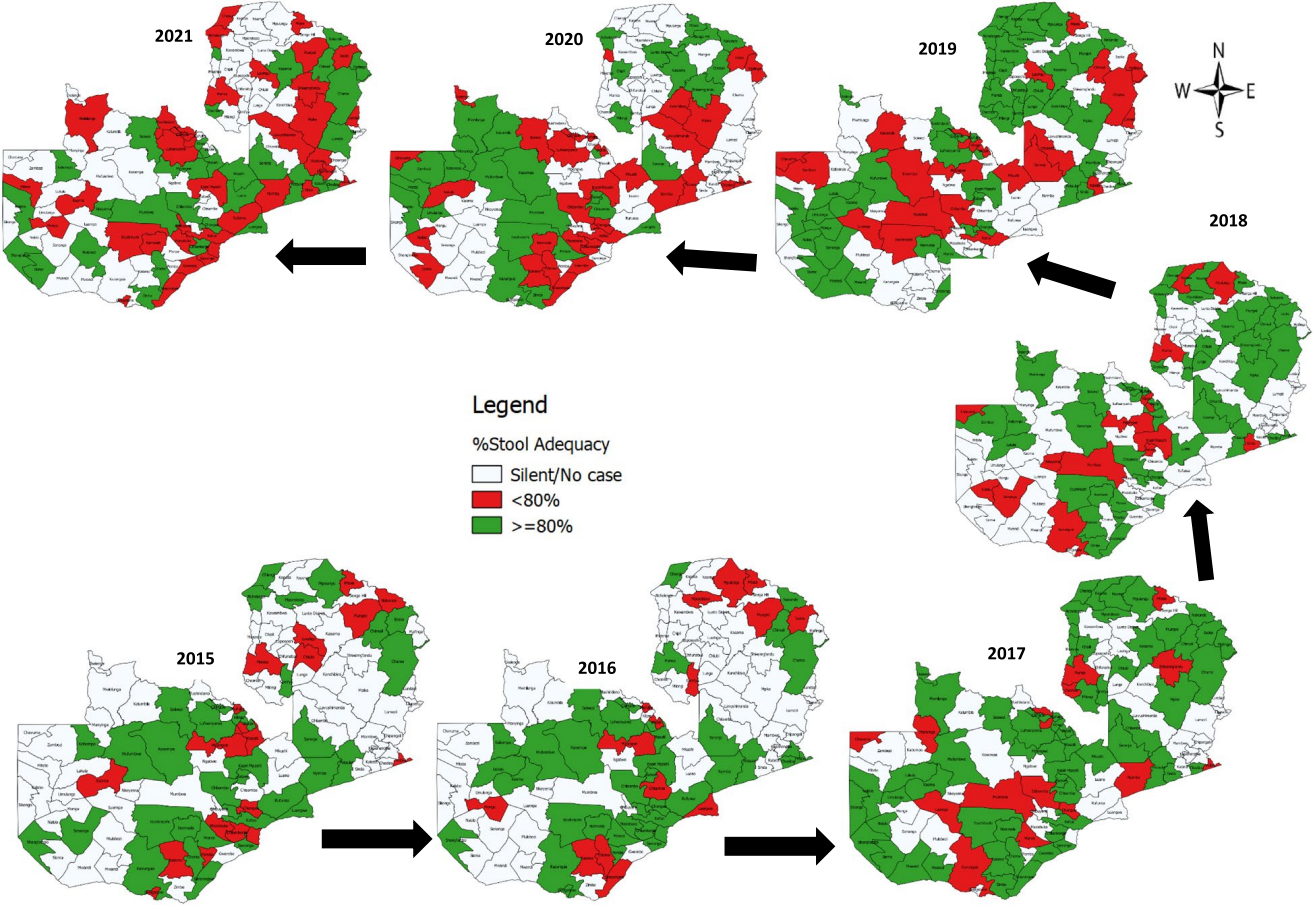
Pattern of stool adequacy rate performance of AFP cases by districts in Zambia from 2015–2021

In terms of stool adequacy, the average stool adequacy over the seven-year period was 83% but this consistently declined from 86.8% in 2015 to 70.9% in 2021 (Fig. 4). At the subnational level (districts), only 46.4% of districts that reported AFP cases over the seven-year period on average achieved the stool adequacy of ≥ 80%. This was much prominent in the year 2020 and 2021 with 33% and 36% of districts achieving the stool adequacy of ≥ 80%, respectively (Fig. 5). For example, only 0.9% (1) rural district consistently achieved this indicator over the seven-year period, but this increased slightly to 3% (4) districts in the last three years (2019–2021) with 75% (3) and 25% (1) of them being rural and urban districts, respectively. Similarly, about 7% (8) of the districts, all rural, consistently did not achieve this indicator over the sever-year period. This proportion however, increased to 31% (36) in the last three years (2019–2021) consisting of 89% (32) rural and 11% (4) urban districts, respectively. After considering only those districts that reported ≥ 5 AFP cases, the proportion reduced to 27% in the last three years with 71% rural and 29% urban districts. This highlights an important surveillance gap that need action.

**Fig. 6:**
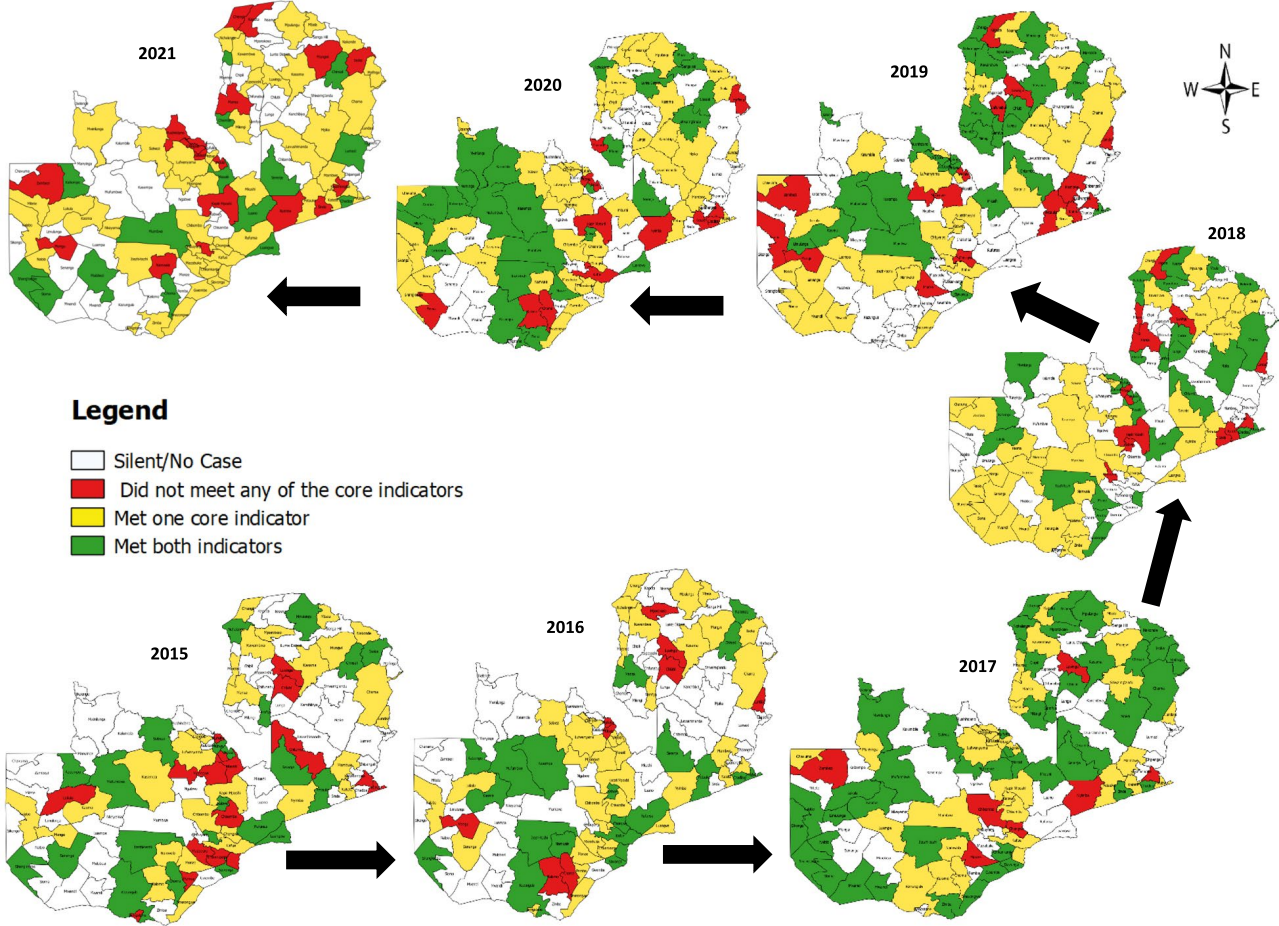
Pattern of achievement of two core AFP surveillance indicators by districts in Zambia from 2015–2021

The proportions of districts that concurrently achieved the two core surveillance indicators (≥ 2/100,000 < 15 years old population and ≥ 80% stool adequacy) declined consistently far below the recommended ≥ 80% target over the seven-years period ranging from 40% in 2017 to 13% in 2021 (Fig. 6). At the subnational level, while no district consistently achieved the two core indicators over the seven-year period, only two rural districts consistently achieved these indicators over the past three years (2019–2021). However, about 13% (15) of the districts did not consistently achieve these two indicators over the seven-year period and this proportion increased to 42% (49) over the past three years (2019–2021) consisting of 90% (44) rural and 10% (5) urban districts. Similarly, after considering only those districts that reported ≥ 5 AFP cases, the proportion reduced to 34% but still substantial in the last three years consisting of 86% rural and 14% urban districts.

The proportion of AFP cases notified within 7 days of onset of paralysis were below the recommend 80% ranging from 78% in 2015 to 58% in 2021 implying a possible late detection of AFP cases. However, most AFP cases (≥ 80% on average) were promptly investigated within 48 h of notification.

**Fig. 7:**
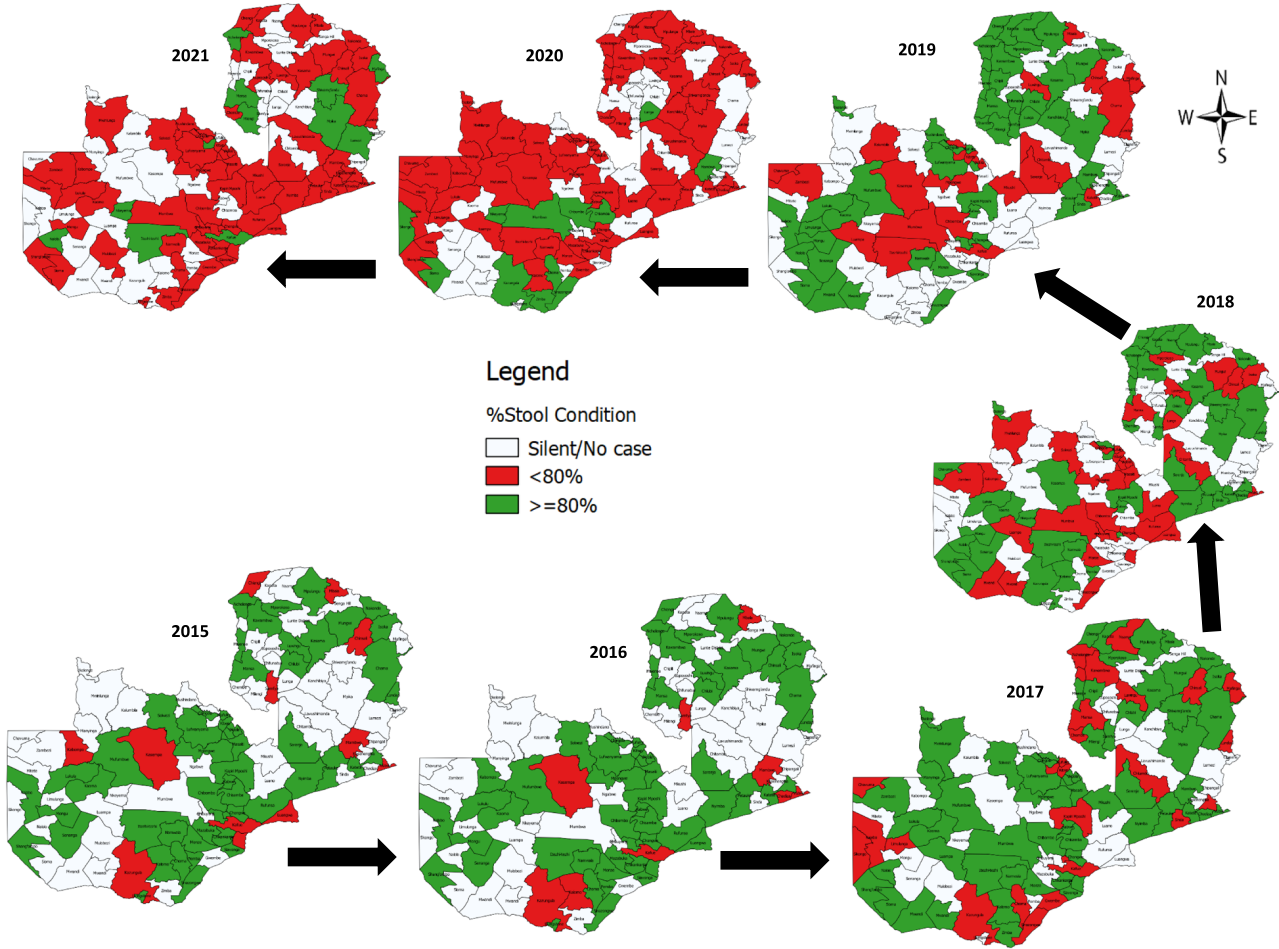
Pattern of % stool sample arriving in good condition in the WHO-accredited lab by districts in Zambia from 2015–2021

Sample transportation to the WHO national accredited polio laboratory has been good. On average AFP stool sample arriving in the laboratory within three days of collection of second stool sample was above the recommend ≥ 80% and range from 94% in 2015 to 90% in 2021. The stool samples arriving in the laboratory in a good condition has been good but consistently declined from 86% in 2015 to 70% in 2021 below the recommended average of ≥ 80%. At the subnational level, the proportion of districts that reported AFP cases with two stool samples arriving in good condition at the WHO-accredited laboratory were consistently far below the recommended ≥ 80% over the seven-year period and ranges from 51% in 2016 to 11% in 2021 (Fig. 7). Surprisingly, no district over the seven-year period or in the past three years (2019–2021) consistently achieved this indicator. However, about 14% (16) of the districts, all rural, consistently did not achieve this indicator over the seven-year period and this proportion increased to 42% (49) in the past three years (2019–2021) with 92% (45) and 8% (4) of them being rural and urban districts, respectively. Similarly, after considering only those districts that reported ≥ 5 AFP cases, the proportions reduced to 35% in the last three years consisting of 86% rural and 14% urban districts.

The proportion of AFP cases with 60-days follow-up evaluation has consistently been below the recommended ≥ 80% over the years and range from 14% in 2015 to 15% in 2021.

## Discussion

This study evaluated the AFP surveillance indicators performance in Zambia from 2015–2021 before the declaration of wild polio virus outbreak in the Southern African region in 2022. In term of demographic and clinical variables, half of the reported AFP cases were < 5 years, more than half had fever at onset of paralysis, and had received three or more doses of oral polio vaccine either via the routine or SIAs. In term of AFP surveillance indicators, we found high NP-AFP rate with subnational (districts) level variations, good stool adequacy rate that declined below the recommended overtime, timely AFP stool sample arrival in the laboratory with moderate proportion of stool samples arriving in good condition, and suboptimal 60-days follow-up evaluation of reported AFP cases. These findings are important because they highlight the strengths and areas to improve to ensure the country maintains its’ polio free status despite the ongoing transmission of wild and circulating vaccine derive polio viruses in the neighbouring countries.

The findings that most (51%) reported AFP cases were < 5 years is significant and implies that the surveillance system could have detected WPV or cVDPV (if it was present) since poliomyelitis commonly affect children within this age group [20]. Similarly, other studies that evaluated AFP surveillance systems also found that most AFP cases were in children less than 5 years in Nigeria (82%) [2], Ghana (76%) [3], Ethiopia (68%) [21], Iran (53%) [22], Bangladesh (54%) [23], and Democratic Republic of the Congo (85%) [24]. However, a previous study in Zambia found that 63% of AFP cases were in the age group 10–15 years [25] but this could be attributed to a significant proportion of cases with undocumented age in their results. The findings of higher proportion of AFP cases being males was also found in previous studies [3, 21, 23].

The analysis indicated that of the reported AFP cases, 69% had fever at onset of paralysis, 42% had asymmetric paralysis, 68% had progression of paralysis within three days, and 53% had received ≥ 3 doses of oral polio vaccines. These signify good documentation of clinical history and immunity profile of reported AFP cases which are important to improve surveillance system performance. For stance, these findings could assist the surveillance system to timely detect a possible “hot” AFP case (a case more likely to be a WPV) [2]. An AFP case must have any three of these to be classified a “hot” case: < 5 years old, < 3 doses of OPV, be clinically compatible (fever at onset, asymmetric paralysis, and rapid progression of paralysis < 3 days), from a high-risk group/area (nomads, refugee camp or a migrant, hard-to-reach due to security), and from a polio free area [2, 20, 26].

WHO criteria for polio-free certification require the detection and investigation of all cases of non-polio AFP in the population < 15 years old. Annually polio-free regions must detect at least 2 AFP cases per 100,000 children aged < 15 years old. The findings indicated that, the average annualised non-polio AFP rate over the seven years was 4.40 per 100,000 population of < 15 years of age with 6.4 in 2015 and 4.1 in 2021, which are far above the WHO recommended value of 2.0 per 100,000 population of < 15 years of age [13] and similar to findings of previous studies [1, 3, 4, 8, 21–23, 27] with higher average annualised non-polio AFP rates reported in Nigeria (9.1) [2] and Democratic Republic of the Congo (4.8) [24]. It is worth mentioning that the country achieved the NP-AFP rate far above the WHO recommended standard value [13] each year for the seven-year study period. This highlights the sensitivity of the surveillance system and likelihood of detecting a poliovirus in the country if it is reintroduced. However, at the subnational level (Districts), there were few districts that did not achieve their targets while others were silent (reported no case in a year when they supposed to report). These silent districts could be due to knowledge gaps, lack of subnational AFP data use for action, and or lack of funding to support AFP surveillance. These gaps could result in a missed opportunity to detect ongoing poliovirus transmission in those districts and highlights the need to continue to support and strengthen AFP surveillance at all levels.

Although the findings on stool adequacy rate had been maintained above the recommended ≥ 80%, the analysis showed drastic declined in 2020 and 2021. While this could partially be attributed to the impact of Covid-19 pandemic as indicated by a recent study [27], a stratified analysis by districts indicated that the proportion has consistently been blow 65% with an average of 46% over the seven-year period. A similar trend of decline in stool adequacy rate was observed between 2012–2020 in other Southern African countries such as Botswana, Mozambique, Namibia and Zimbabwe [27, 28]. Our study findings may imply knowledge and logistics gaps resulting in low AFP cases awareness levels, late reporting of cases, inadequate logistics especially for sample collection, and poor maintenance of reverse cold chain during sample collection and transportation.

This study highlighted a consistent decline in the proportion of districts achieving both the NPAFP rates and stool adequacy targets over the seven-year period ranging from 40 to 13% which is far below the recommended ≥ 80%. This gap was much wider at the subnational level and may negatively impact timely detection of silent poliovirus transmission, effective outbreak response strategies and consequently may results in wider spread of the poliovirus. Based on the Global Polio Surveillance Action Plan 2022–2024 [19], we recommend the following to improve these indicators: 1) Using the current epidemiology and health-seeking behaviours in country to prioritise active surveillance sites including traditional/faith-based healing sites, 2) Regularly review active surveillance sites visits for timeliness and completeness per planned prioritisation visits and ensure field officers are accountable for these sites visits, 3) Regularly identify underperforming areas and blind spots for action via mapping of hard-to-reach and special populations, 4) Identify barriers to early case detection for targeted action via regular monitoring of timeliness of detection, tacking of subnational delays and disaggregated data analysis, 5) Prioritise active surveillance visits to areas with high-risk for poliovirus importation, outbreaks or possible missed transmissions, and 6) Develop, implement and monitor pragmatic surveillance enhancement plan activities based on gaps identified.

While AFP cases were promptly investigated upon notification, early detection of AFP cases was low. There was a consistent decline from 78% in 2015 to 58% in 2021 in the proportion of cases notified within days of onset of paralysis which is far below the recommended ≥ 80%. Our result is consistent with a similar decline observed in early detection of AFP cases in all East and Southern African countries between 2012–2020 [27, 28]. This finding may imply poor AFP health seeking behaviour where parents prefer to visit the traditional or faith healers first before the orthodox health facilities, low community or health worker sensitisation, and inadequate active case search in communities especially at the traditional and faith healing centres.

The results of the analysis indicated that while the percentage of stool samples arrival in the laboratory ≤ 3 days had been consistently above the recommended ≥ 80%, the proportion arriving in good condition declined over time during the seven-year period below the recommended ≥ 80%. A recent analysis of the impact of the covid-19 pandemic on the AFP surveillance in the East and Southern Africa regions highlighted increased proportions of stool samples arriving in the laboratory within 3 days but decreased in the proportions of samples arriving in good condition [27]. These findings are important since they highlight that sample transportation may not the problem but sample collection, documentation, reversed cold chain, and logistics may be the major challenges.

Follow up evaluation of AFP cases after 60-days of onset of paralysis is very important for the surveillance system. It provides the opportunity to determine the presence of residual paralysis and sufficient evidence to support decision to identify and classify reported AFP cases ‘compatible’ with polio or ‘discarded’ (non-polio). Our study indicated that only 14% of AFP cases on average had 60-days follow-up evaluation done in the seven-year period with the maximum being 28%. Considering the late detection of cases and suboptimal stool adequacy at district level over the study period, this finding may imply that final classification of discarded (non-polio) cases might be done possibly without sufficient information which could hinder the identification of weak surveillance areas and missed opportunity of detecting poliovirus transmission.

The practical implication of the surveillance gaps identified in this study is that silent poliovirus transmission and subsequent expansion of transmission in the country may go unnoticed. To mitigate these challenges, we recommend the following: 1) Regular analysis and tracking of surveillance indicators including health-seeking behaviours of care-seekers and social mapping for action, 2) Undertake regular supportive supervisory visits to all surveillance sites including the traditional and faith-based healing sites, 3) Regular surveillance refresher trainings and review meetings including community base volunteers and traditional/faith-based healers, 4) Printing and distribution of surveillance information, education and communication (IEC) materials at all sites to increase sensitivity, 5) Adequate supply of case investigation kits and support care-givers and care-seekers in stool collection, and 6) Integrate surveillance activities including active case search, community-based surveillance, supportive supervision, reviews and sample transportation [19].

In conclusion, our study revealed that Zambia AFP surveillance system is performing well in meeting the non-polio AFP rate which exceeded the WHO target and moderate stool adequacy over the seven-year period (2015–2021). However, the national aggregated data obscured district level performance in terms of early AFP case detection, stool adequacy, and 60-days follow-up evaluations which leave the county at risk of failing to detect any possible transmission of poliovirus. Considering the ongoing poliovirus transmission in the subregion, we highly suggest intensive support to enhance the surveillance system in terms of funding, building capacity of all surveillance focal points including the sub-national levels, supportive supervision, and logistics for sample collection and transportation, to sustain the polio free status of the country.

## Data Availability

The datasets used in this current study is available from the corresponding author on reasonable request.

## Abbreviations

AFP: Acute Flaccid Paralysis
GPEI: Global Polio Eradication Initiative
WHO: World Health Organization
EPI: Expanded Program on Immunisation
WPV: Wild Polio Virus
IDSR: Integrated Disease Surveillance System
MOH: Ministry of Health
ZNPHI: Zambia National Public Health Institute
DSO: Disease Surveillance Officer
UTH: University Teaching Hospital
NPEC: National Polio Expect Committee
POLIS: Polio Information System
QGIS: Quantum Geographical Information System
OPV: Oral Polio Vaccine
